# Land Management Practices Associated with House Loss in Wildfires

**DOI:** 10.1371/journal.pone.0029212

**Published:** 2012-01-18

**Authors:** Philip Gibbons, Linda van Bommel, A. Malcolm Gill, Geoffrey J. Cary, Don A. Driscoll, Ross A. Bradstock, Emma Knight, Max A. Moritz, Scott L. Stephens, David B. Lindenmayer

**Affiliations:** 1 The Fenner School of Environment and Society, The Australian National University, Canberra, Australian Capital Territory, Australia; 2 Centre for Environmental Risk Management of Bushfires, University of Wollongong, Wollongong, New South Wales, Australia; 3 Centre for Mathematics and its Applications, The Australian National University, Canberra, Australian Capital Territory, Australia; 4 Ecosystem Sciences Division, Department of Environmental Science, Policy and Management, University of California, Berkeley, California, United States of America; Monash University, Australia

## Abstract

Losses to life and property from unplanned fires (wildfires) are forecast to increase because of population growth in peri-urban areas and climate change. In response, there have been moves to increase fuel reduction—clearing, prescribed burning, biomass removal and grazing—to afford greater protection to peri-urban communities in fire-prone regions. But how effective are these measures? Severe wildfires in southern Australia in 2009 presented a rare opportunity to address this question empirically. We predicted that modifying several fuels could theoretically reduce house loss by 76%–97%, which would translate to considerably fewer wildfire-related deaths. However, maximum levels of fuel reduction are unlikely to be feasible at every house for logistical and environmental reasons. Significant fuel variables in a logistic regression model we selected to predict house loss were (in order of decreasing effect): (1) the cover of trees and shrubs within 40 m of houses, (2) whether trees and shrubs within 40 m of houses was predominantly remnant or planted, (3) the upwind distance from houses to groups of trees or shrubs, (4) the upwind distance from houses to public forested land (irrespective of whether it was managed for nature conservation or logging), (5) the upwind distance from houses to prescribed burning within 5 years, and (6) the number of buildings or structures within 40 m of houses. All fuel treatments were more effective if undertaken closer to houses. For example, 15% fewer houses were destroyed if prescribed burning occurred at the observed minimum distance from houses (0.5 km) rather than the observed mean distance from houses (8.5 km). Our results imply that a shift in emphasis away from broad-scale fuel-reduction to intensive fuel treatments close to property will more effectively mitigate impacts from wildfires on peri-urban communities.

## Introduction

Peri-urban communities in fire-prone regions around the world are at increasing risk from unplanned fires (wildfires) because of population growth [1], [2], [3] and climate change [4], [5], [6], [7]. The potential consequences of these factors were illustrated by recent major wildfires in California (26 deaths, 3361 houses lost) [8], Russia (54 deaths, circa. 2000 houses lost) [9] and Australia (173 deaths, 2133 houses lost) [10]. The behaviour of wildfires is primarily determined by weather, terrain and fuel [11]. Fuel in vegetation is often the easiest of these to manipulate [12]. Thus, there have been moves to increase the area of fuel reduction in many fire-prone regions [10], [13], [14].

Common fuel-reduction treatments employed in fire-prone landscapes are clearing, prescribed burning, grazing and mechanical removal of biomass (e.g., thinning) [6], [12], [13], [15]. These treatments are often undertaken at broad-scales and distant from peri-urban communities. For example, in the United States of America, 89% of all fuel-reduction treatments undertaken on federal lands were >2.5km from the wildland urban interface [13]. Fuel treatments can be expensive [13] and can have undesirable health [16] and environmental [17] impacts (although not in all cases [18]). Yet, evidence that these treatments mitigate impacts on peri-urban communities from wildfires remains extremely limited [1].

Houses are a critical asset to protect during wildfires because most wildfire fatalities occur among people evacuating late from, sheltering in, or defending them [19]. Houses are destroyed during wildfires when exposed to flames in adjacent fuel, radiant heat from nearby fuel (≤40m) [20], or airborne embers and firebrands originating in nearby and distant fuel (typically<10 km) [21], [22]. However, the relative importance of these different fuels—and therefore the relative effectiveness of different fuel treatments in protecting houses—have not been examined empirically. This is because wildfires are a difficult phenomenon to study [1], [23]. Large, destructive fires cannot be lit experimentally, house loss during wildfires is often aggregated, preventing replication of landscape-scale variables, and adequate pre- and post-fire data are not always available. Thus, there are few wildfires that lend themselves to empirical research on the effects of the full range of fuel treatments on house loss.

Wildfires in south-eastern Australia in 2009 destroyed a large population of houses in landscapes with a mix of housing densities, terrains and fuel types, and occurred in landscapes where there were adequate pre- and post-fire data. These wildfires therefore provided a rare opportunity to robustly quantify and compare the effectiveness of different fuel types and different fuel treatments on house loss during wildfire.

## Results

To quantify the relative effects of different fuels on house loss we sampled 499 houses and at each house recorded 24 potential explanatory variables representing the three principal drivers of fire behaviour (i.e. weather, terrain and fuel) [11]. We sampled extremes in several of these variables not achieved in previous studies. For example, the Forest Fire Danger Index (FFDI) [24] at sampled houses ranged from 5 to 189, slope ranged from 0.3 to 22.6°, the percent of cleared land upwind from houses ranged from 0 to 100% and the percent of land prescribe-burnt within 5 years upwind from houses ranged from 0 to 36.4%.

### A model to predict house loss

**Figure 1:**
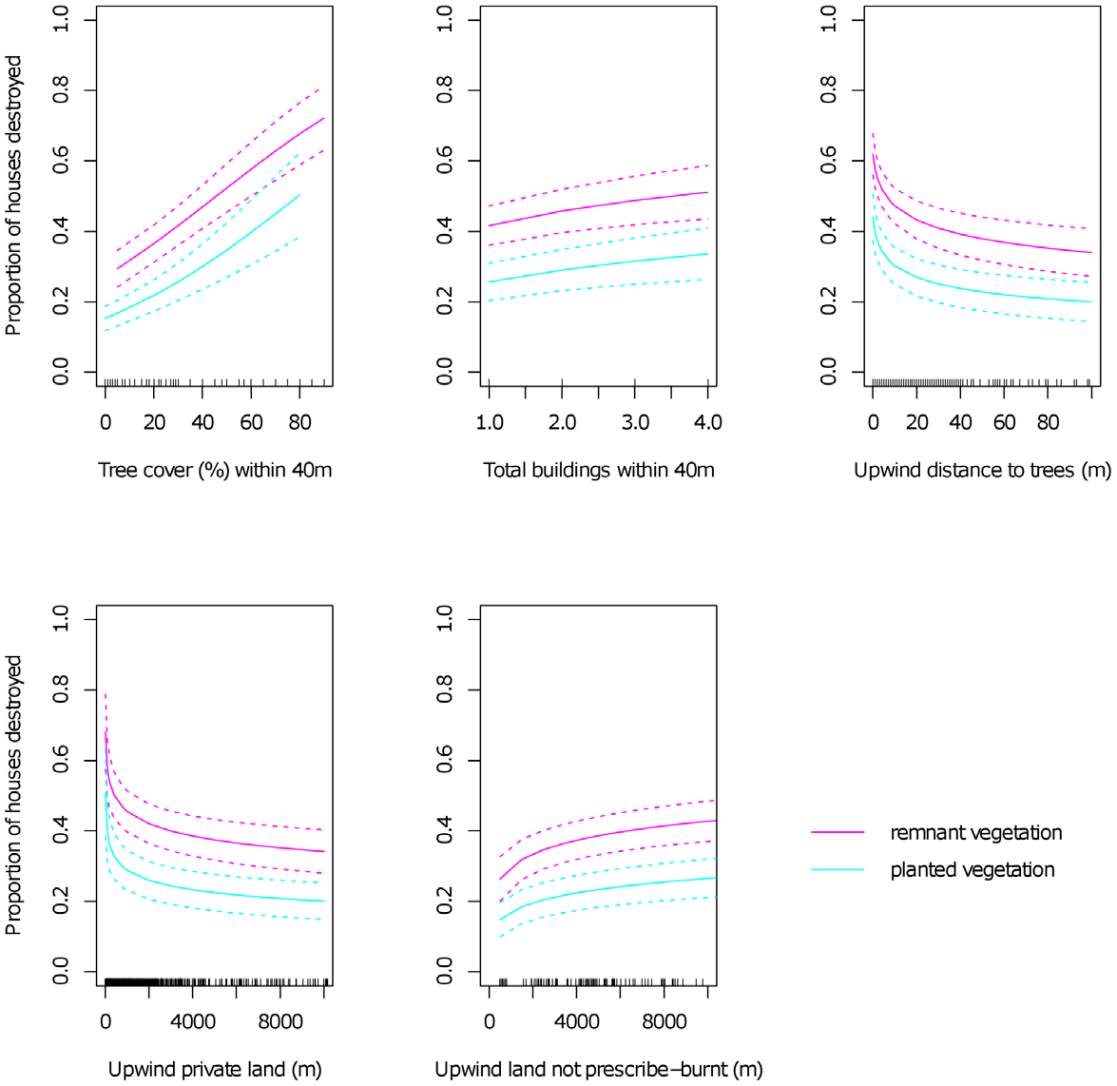
Individual effects (mean ± s.e.m.) of fuel variables in the logistic model used to predict the proportion of houses lost during wildfire. Each prediction was made with the other significant explanatory variables held at their means and FFDI fixed at 100, which is the value above which 64% of houses have been destroyed in wildfires in Australia [26]. Magneta (pink) lines are predictions for vegetation within 40 m of houses that is predominantly remnant native vegetation and cyan lines are predictions for vegetation within 40 m of houses that is predominantly planted vegetation.

A logistic regression model we selected to predict house loss contained eight significant explanatory variables (Table 1). This model indicated that a greater proportion of houses were lost where: there was a higher % cover of trees and shrubs within 40 m; the vegetation within 40 m was dominated by remnant native (rather than planted) vegetation; there were more buildings within 40 m; groups of trees or shrubs were closer in the upwind direction; forest burnt within 5 years in the upwind direction was distant rather than close; and houses were closer to public land (had less private land) in the upwind direction (Figure 1). In the best alternative logistic model we identified, variables representing the amount of land that is not State Forest and the amount of land that is not National Park replaced the amount of private land upwind from houses (together the former are negatively correlated with the latter). This alternative logistic model indicated that, other things being equal, houses were at similar risk when they occurred close to either National Park or State Forest. None of the interactions we tested (see Materials and Methods) were significant in the selected model.

**Table 1:** The selected logistic regression model used to predict the proportion of houses lost during the sampled wildfires.

Variable	Coefficient ± s.e.m	*P*(>|z|)
Intercept	−5.687±1.073	0.000
Tree and shrub cover (%) within 40 meters (m)	0.022±0.006	0.000
Log_10_ (FFDI)	1.062±0.3076	0.000
Log_10_ (amount of land not burnt within 5 years (m))	0.565±0.216	0.001
Vegetation type within 40 m (planted)	-	-
Vegetation type within 40 m (remnant)	0.726±0.246	0.003
Log_10_ (amount of private land (m)+1)	−0.479±0.199	0.016
Log_10_ (distance to nearest trees and shrubs (m)+1)	−0.574±0.191	0.003
Log_10_ (buildings within 40 m)	0.963±0.483	0.046
Autocovariate (spatial autocorrelation)	4.800±1.110	0.000

Significant explanatory variables, their coefficients and *P*-values in the logistic model selected to predict the (logit or log-odds) proportion of houses destroyed during wildfire. Vegetation type is a categorical variable with ‘planted’ being the reference level. The autocovariate represents spatial autocorrelation between neighbouring houses.

The selected logistic regression model included several variables in addition to fuel that also affect fire behaviour. Other things being equal, weather had a strong effect, with a greater proportion of houses lost at higher levels of temperature and wind speed and lower levels of relative humidity (measured using FFDI). We included an “autocovariate” [25] in the selected logistic regression model to account for spatial autocorrelation between houses within 1 km of each other (see Materials and Methods). No variables representing terrain were significant in the selected model.

A Hosmer–Lemeshow test for the selected logistic model indicated that observed house loss was not significantly different from predicted house loss (*P* = 0.487). The area under the Receiver Operating Characteristic Curve (AUC) indicated that the fitted logistic model correctly discriminated between burnt and unburnt houses 80% of the time.

Predictions from the fitted logistic model indicated that reducing fuel could substantially reduce the number of houses destroyed during severe wildfires. With variables representing fuel held at observed minimum loads (i.e., 10% cover of planted trees and shrubs within 40 m from houses, 100 m to the nearest trees and shrubs in the upwind direction, 500 m to forest burnt ≤5 years ago in the upwind direction and no buildings within 40 m); and other variables fixed at their means (i.e., FFDI, the distance to public land and the covariate representing spatial autocorrelation), we predicted that 4.6% (±1.9% s.e.m) of all houses would be destroyed. Thus, under otherwise average conditions observed during these wildfires, minimizing key fuels at every house could potentially reduce the percent of houses destroyed from the observed value of 35.0% to a predicted mean value of 4.6% (±1.9% s.e.m). This equates to a reduction in the number of houses lost of 76%–97% (95% confidence interval). However, this level of fuel management is unlikely to be realised at all houses for reasons we outline in the Discussion.

### The relative effects of fuel variables on house loss

We used mean predictions from the selected logistic model to examine the relative effects of different fuels on house loss (Figure 1). Predictions for each fuel variable were made with the other significant explanatory variables held at their means, but with FFDI held at 100. This is the value for FFDI above which 64% of houses have been destroyed by wildfires in Australia [26] and the value for FFDI that invokes the highest level of public warning in fire-prone regions of Australia. We predicted that reducing remnant native vegetation around houses (within 40 m) from 90% cover (the observed maximum) to 5% cover reduced the likelihood of house loss by 43%. That is, every 10% reduction in remnant native vegetation cover around houses reduced the likelihood of house loss by approximately 5%. Thirty eight percent fewer houses were destroyed if surrounded (within 40 m) by predominantly planted vegetation rather than predominantly remnant native vegetation. Twenty six percent fewer houses were lost if further (100 m) from the nearest group of trees or shrubs in the upwind direction, compared with houses adjacent (0 m) to groups of trees or shrubs in the upwind direction. Compared with houses located 10 m from public forest, 14% fewer houses were lost if 200 m from public forest, and 26% fewer houses were lost if 2 km from public forest (the average distance between houses and public forest). On average, 15% fewer houses were lost if prescribed burning within 5 years was undertaken 0.5 km upwind from houses (the nearest distance between houses and prescribed burning), rather than 8.5 km upwind from houses (the average distance between houses and prescribed burning). We predicted a 3% increase in the number of destroyed houses with every additional building or shed located within 40 m.

## Discussion

We predicted that modifying key fuels could substantially reduce house loss during wildfires that burn in extreme fire weather conditions. Many deaths occur among people sheltering in houses during wildfires (69% of lives lost during the 2009 wildfires examined here were in houses [10]), so managing key fuels could also save considerable lives.

### The relative effects of fuel treatments on house loss

We found that modifying fuel closer to houses was a more effective way to reduce house loss than modifying fuel distant from houses. In severe fire weather (FFDI = 100), we predicted that reducing trees and shrubs from 90% cover to 5% cover within 40 m of houses could potentially reduce house loss by an average of 43%, making this the single most effective fuel treatment that we measured. We predicted that conversion from predominantly remnant to predominantly planted vegetation within 40 m from houses could reduce house loss by 38%. Increasing the upwind distance from houses to groups of trees and shrubs from zero to 100 m would reduce the number of houses lost by an average of 26%. The distance between houses and public forest had a similar effect. Twenty six percent fewer houses were lost 2 km from public land (the mean observed distance) compared with houses adjacent to public land. We predicted that an average of 15% fewer houses were destroyed when prescribed burning was undertaken 0.5 km from houses (the minimum distance we observed), compared with 8.5 km from houses (the mean distance we observed). One less building within 40 m from a house reduced the likelihood of destruction by an average of 3%, making this the least influential fuel variable in the selected logistic regression model.

Our finding that fuel management close to houses was more effective than fuel management further from houses can be explained by the behaviour of embers and radiant heat—the principal causes of house loss during wildfires [20], [22], [27]. The density of airborne embers [21] and the amount of radiant heat [20] are greatest closer to the fuel source, which is consistent with our results that fuel and fuel treatments closer to houses were more strongly associated with house loss. The reduction of fuel close to houses also increases ‘defensible space’, or the area around houses in which suppression is most likely to be successful [23].

### Prescribed burning and house loss

Prescribed burning is a widely employed fuel treatment in many regions [12], [13] and many commentators identified this as the key fuel treatment contributing to house loss in our study area. Although there was relatively limited prescribed burning in many parts of our study area, stratifying by this variable enabled us to sample houses with between 0% and 36.4% of the landscape burnt within 5 years in the upwind direction to the 2009 wildfire boundary. Within this range of variation, we found the effect of prescribed burning within 5 years was greatest closer to houses (Figure 1). This pattern is consistent with our results across all fuel variables (Figure 1). It is also noteworthy that prescribed burning was not a significant explanatory variable in any of the feasible logistic regression models when it was measured as the percentage of the landscape treated in the upwind direction from houses to the nearest 2009 wildfire boundary (rather than upwind distance from houses to the closest prescribed burning). This suggests that the proximity to houses of prescribed burning is more important than the total percentage of the landscape that is prescribe-burnt. These results are consistent with previous research indicating the effects of prescribed burning can diminish within a short period of time (2–6 years) [12], [28], [29] and in severe fire weather conditions [8], [12], [30], which are the conditions when most houses are destroyed [26]. Our results therefore indicated that prescribed burning—when executed at the scale observed in this study—was most effective when undertaken close to houses and at least every 5 years.

It is argued [10], based on relationships between prescribed burning and changes to the incidence and extent of wildfires [31], [32], that prescribed burning can make control and suppression of wildfires before they reach houses more effective if executed in larger units and over a larger percentage of the landscape than observed in this study. However, it remains untested whether this strategy is effective in the extreme weather conditions. It is also important to note that the extent of prescribed burning that is feasible in many landscapes, including our study area, is restricted because of the number of days per year in which weather conditions are suitable and/or the proximity of public land to infrastructure [12].

### Conclusions

Devastating wildfires provide a window into conditions that may become more common in the future [1], [2], [3], [4], [5], [6] and therefore represent important learning opportunities for decision-makers. The typical response to destructive wildfires is to increase the total area of land that is fuel-reduced [10], [13]. Our results instead indicate that a shift in emphasis from broad-scale fuel-reduction treatments to intensive fuel treatments close to houses will more effectively mitigate impacts from wildfires on houses. This result is consistent with observations that the density of airborne embers and amount of radiant heat (the principal causes of house loss during wildfires) are greatest closer to the fuel source. This suggests that the actions of private landholders, who manage fuel close to houses, are extremely important when reducing risks to houses posed by fuel. Our results are based on data collected at wildfires in south-eastern Australia. While it has been speculated that these conclusions apply to other regions around the world [13], the broader applicability of our results can only be confirmed with sampling across a broader range of fuel types and climates.

Although our results indicated that risks posed to peri-urban communities by severe wildfires can be reduced by effectively managing fuel, these risks cannot be eliminated by managing fuel alone. Fuel treatments can be expensive [13] and can have undesirable health [16] and environmental [17] impacts (but not in all cases [18]). Therefore, intensive fuel-reduction is not always an appropriate strategy to reduce risk posed by wildfire. Weather strongly influenced the effect of fuel variables (Table 1), hence other measures not accounted for here (e.g., architectural solutions, education of residents, suppression effort, safer places, early evacuation) [22], [33], [34], [35] must remain part of a strategy to mitigate increasing risks to communities from wildfires.

Overall our results clearly imply that fuel close to housing plays a key role in house loss during wildfire, so fuel management should be considered as part of a strategy to mitigate increasing risks to peri-urban communities from wildfires. Future impacts from wildfires will be reduced, and the negative effects of fuel treatments avoided, if new peri-urban developments in fire prone regions are restricted to areas where there is adequate separation between high fuel loads and houses.

## Materials and Methods

### Study area and stratification

Houses were sampled within the boundaries of three wildfires that ignited in the State of Victoria, south-eastern Australia on 7 February, 2009. These fires were known as: the Kilmore East fire, which burnt 125,383ha, destroyed 1242 houses and claimed 119 lives; the Murrindindi fire, which burnt 43,159ha, destroyed 538 houses and claimed 40 lives [10]; and the Churchill fire which burnt 25,861ha, destroyed 145 houses and claimed 11 lives (Figure S1). The wildfire boundaries were as mapped in the FIRE_SEV09 GIS shape file provided by the Victorian Department of Sustainability and Environment (DSE). We stratified the study area by the three principal drivers of wildfire behaviour: weather, terrain and fuel [11]. Weather (measured using FFDI), ranged from 5 to 189. Slope of the terrain at each house ranged from 0.3° to 22.6°. Fuel, measured as % burnt upwind from houses within 5 years to the 2009 wildfire boundary and as the % of the landscape cleared upwind from houses to the 2009 wildfire boundary, ranged from 0% to 36% and 0% to 100% respectively.

### Response variable

Our response was a binary variable representing house loss (intact or destroyed). To sample houses we allocated 499 points randomly to the study area in a Geographical Information System (GIS) in numbers proportional to the area of each stratum. We then selected the nearest house to each point using fine-scale (35 cm to 50 cm pixel resolution) orthorectified aerial imagery in the visible spectrum taken between 1 and 37 months prior to the fires. Our sampling of houses was blind in the sense that we did not know which had been destroyed. Several variables were measured within a circle with a 40 m radius from the centroid of each house (Table S1). To increase the likelihood of independence between responses we did not sample houses when these circles overlapped, instead choosing another random point until a non-overlapping house with 40 m circle was located. We recorded damage to each sampled house (intact or destroyed) based on a visual inspection of the house using fine-scale (15 cm pixel resolution) orthorectified imagery in the visible spectrum taken 17–23 days after the fires. We judged a house as destroyed if at least part of the roof had visibly collapsed or incinerated and judged a house as intact if the roof remained. In all cases this distinction was clear, which is consistent with on-ground observations by Wilson and Ferguson [33] who rejected using a continuous or ordinal variable to categorise house damage because virtually all houses affected by wildfire in their study had either been destroyed or sustained only superficial damage.

### Potential explanatory variables

We recorded 24 potential explanatory variables at each house reflecting the three drivers of fire behaviour (i.e. weather, terrain and fuel) [11].

**Weather** conditions were measured with the Forest Fire Danger Index (FFDI) because house loss in Australian fires exhibits a higher correlation with this index rather than any of its individual components (i.e., wind, temperature, relative humidity and drought factor) [26]. FFDI was calculated using the formula [24]

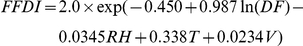
where, *DF* is drought factor, *RH* is relative humidity (%), *T* is air temperature (°C) and *V* is wind speed (km hr^−1^). Weather variables used to calculate FFDI (and to calculate wind direction for some fuel variables in Table S1) were derived from half-hourly data recorded at the closest weather station to each house [36]. We were advised by the Australian Bureau of Meteorology that these were the most reliable weather data available for our purpose. The estimated time that fire impacted on each house was estimated from fire progression maps provided by DSE for the Kilmore East and Murrindindi fires and a fire progression map prepared by the Victorian Country Fire Authority for the Churchill fire [10].

**Terrain** was measured as slope, topographic position and aspect (Table S1).

**Fuel** was measured (a) within 40 m of the centroid of each house, which is the approximate maximum distance that radiant heat is likely to ignite a wooden structure [20], (b) as a percentage of the landscape along a single transect in the upwind direction from each house to the nearest 2009 wildfire boundary, which ensured there was little overlap between measurements taken for different houses at this scale, and (c) as the distance from each house to the fuel variable in the upwind direction (Table S1). We could not measure the distance from each house to several fuel variables (public land, previous burning within 10 years, logging within 30 years, National Park, State Forest) because they did not always occur between sampled houses and the 2009 wildfire boundary in the upwind direction. If we excluded houses that did not have all of these fuel variables in the upwind direction then this would bias the sample (only 13 of the 499 sampled houses had all of the measured fuel variables in the upwind direction to the 2009 wildfire boundary). We therefore measured the amount of land upwind from houses that did NOT contain these fuel variables. For example, instead of recording the distance from houses to land burnt within 5 years in the upwind direction, we recorded the amount of land not burnt within 5 years in the upwind direction between each house and the 2009 wildfire boundary. This enabled us to include all randomly sampled houses in the analysis. The % cover of trees and shrubs within 40 m from the centroid of each house was estimated visually on the pre-fire aerial imagery by one person (P.G.). To test the accuracy of this method, we randomly selected 30 houses and compared our visual % cover estimates with estimates for the same houses derived by digitising tree and shrub cover in a GIS. Visual % cover estimates were highly correlated with % cover estimates derived from digitising trees and shrubs (r = 0.95, Pearson correlation coefficient). The mean (± s.e.m.) % cover of trees and shrubs derived from visual estimates (33.2±4.4) was not significantly different from estimates derived from digitising trees and shrubs (32.1±4.0) (*P* = 0.462, 2-tailed t-test).

### Exploratory data analysis

Summary statistics for the continuous potential explanatory variables (see Table S1 for definitions) (mean, range) were: FFDI (48, 5–189), slope (8.5°, 0.3–22.6°), aspect (186°, 25–329°), number of buildings (2, 1–9), % cover of trees and shrubs (30%, 0–90%), distance to nearest tree or shrub (2.6 m, 0–108 m), upwind distance to nearest trees or shrubs (26 m, 0–686 m), upwind distance to nearest block of trees (272 m, 0–3021 m), upwind distance to mapped cleared land (773 m, 0–25121 m), amount of private land (2145 m, 0–15280 m), % cleared (32%, 0–100%), amount of land not burnt for ≤5 years (8848 m, 14–40041 m), % of land burnt ≤5 years ago (2.8%, 0–36.4%), amount of land not burnt for >5–10 years (10985 m, 14–35157 m), % of land burnt >5–10 years ago (0.4%, 0–37.7%), amount unlogged (9107 m, 14–36168 m), % logged (1.7%, 0–32.9%), amount not National Park (7457 m, 14–35157 m) and amount not State Forest (5501 m, 13–24703 m). The % of houses with the different measured fuel variables in the upwind direction within the 2009 wildfire boundary were: trees or shrubs (97%), block of trees (97%), land burnt within 5 years (25%), land burnt within >5–10 years (12%), logged within 30 years (26%), mapped cleared land (94%), public land (75%), National Park (41%) and State Forest (72%). The majority (86%) of the area burnt within 10 years was from prescribed fire, with the remainder burnt from wildfire.

We constructed a correlation matrix using the Pearson correlation coefficient (r) for all pairs of potential explanatory variables to determine those highly correlated (r≥0.7). One pair of potential explanatory variables was highly correlated: distance from houses to the 2009 wildfire boundary and amount of land not burnt for >5 to 10 years (r = 0.83). Only one of each of these variables was included in any model. Several other pairs of variables (amount of land not burnt for ≤5 years, amount unlogged, amount of land that is not National Park, amount of land that is private and amount of land that is not State Forest) were reasonably highly correlated (r = 0.59–0.68), but were included in all models. The following variables had highly skewed distributions and were therefore transformed by log_10_ (*x*) (or log_10_(*x*+1) for variables with zeros) to give them a more symmetrical distribution prior to statistical modelling: upwind distance to nearest trees or shrubs, upwind distance to nearest block of trees, upwind distance to mapped cleared land, amount of land upwind from houses not burnt for ≤5 years and >5–10 years, amount of land upwind from houses that is privately owned, buildings within 40 m of houses and the amount of land upwind from houses that is not National Park or State Forest.

### Statistical analysis

We initially examined relationships between the response and uncorrelated potential explanatory variables using mixed-effects modelling to account for the hierarchical structure of our data (i.e., houses were sampled within three separate fires which ignited at different times of the day and in different regions). That is, there was potential for the effect on houses of the same fire to be more alike than the effect on houses in a different fire. However, initial analyses using Generalized Linear Mixed Modelling (GLMM), implemented using the MGCV library in R [37], indicated that the variable representing the three different fires had a variance component approaching zero (<0.001). That is, the response did not change between fires. Remaining analyses were therefore undertaken using the more parsimonious Generalized Linear Modelling (GLM).

We used GLM with a logit link implemented using the MASS library in R [37] to identify fuel variables that were the best predictors of house loss. We accounted for the influence of weather and terrain by fitting FFDI slope, aspect and topographic position as co-variates during model selection. We included several interaction terms during model selection. To test whether effects of fuel variables varied with weather conditions we included interactions between FFDI and some fuel variables (% of landscape not burnt in the upwind direction, amount of land not burnt in the upwind direction, nearest upwind distance to trees or shrubs, % cover of trees and shrubs ≤40 m from houses). To test whether the effect of slope on the proportion of houses destroyed was influenced by aspect we fit an interaction term between slope and aspect. To test whether there was an interaction between defensible space created by clearing close to houses and broader-scale fuel reduction, we included interaction terms between the % cover of trees and shrubs ≤40 m from houses and the amount of previous burning in the upwind direction from houses.

We chose a model of ‘best fit’ using stepwise selection [38]. We first fitted a full model (with all terms) and then dropped terms sequentially if they did not lower Akaike's Information Criterion (AIC). Following Venebles and Ripley [38] (pp. 175–176), we then dropped any variables from this model if they were not significant (*P*≤0.05) using the traditional analysis of deviance, thus obtaining a more parsimonious result. All predictions were made from this single model of ‘best fit’.

Because many houses in our study area occurred in a clustered spatial arrangement around towns (Figure S1), it follows that there is potential for spatial autocorrelation in our data. That is, if one house is destroyed then neighbouring houses are more likely to be destroyed, which would violate the assumption of independence in our fitted GLM. We tested whether residuals from the fitted GLM were spatially autocorrelated using Moran's I. This test was undertaken using the Ape package in R [37], which is based on the method described by Gittleman and Kot [39]. Moran's I, calculated using the residuals from the selected logistic model, was significantly different (*p*<0.001) to the expected value of Moran's I if the residuals were distributed randomly, leading us to conclude that there is strong evidence for spatial autocorrelation in our data.

To account for this spatial autocorrelation we added an “autocovariate” [25] to the fitted GLM, which is a covariate representing spatial autocorrelation, following the methodology for non-normally distributed data reported in the appendix to Dormann et al. [40]. The autocovariate was scaled from zero (there was no relationship in the response between neighbouring houses) to 1 (the response was identical between neighbouring houses). The autocovariate was calculated using a matrix of neighbours within 1 km of each house. A correlogram of Moran's I indicated that most spatial autocorrelation in the residuals between neighbouring houses occurred within this distance. We added the autocovariate to the fitted GLM and then, again using the method of Gittleman and Kot [39], we confirmed that Moran's I calculated using the residuals in this new model was no longer significantly different to expected if distributed randomly (*p* = 0.845).

We reported goodness of fit for our selected logistic model using the Hosmer-Lemeshow test calculated using the Design library in the R statistical software [37]. We calculated AUC [41] from observed and predicted values for this model using the package Rocr in the R statistical software [37] to determine the probability that true positives rank above false positives. AUC has a value between 0.5 (a discriminating ability no better than chance) to 1 (perfect discriminating ability).

## Supporting Information

Figure S1 **Houses sampled in (A) the Kilmore East Murrindindi wildfires and (B) the Churchill wildfire.** Sampled houses that were intact (clear houses) and destroyed (solid red houses) after the wildfires are illustrated.
(TIF)

Table S1 **Potential explanatory variables recorded for each sampled house.**
(DOC)
