# Supplementary figures and images for: Land Management Practices Associated with House Loss in Wildfires
Source: PLoS One. 2012 Jan 18;7(1):e29212. doi: 10.1371/journal.pone.0029212 (PMC3260958; doi:10.1371/journal.pone.0029212)

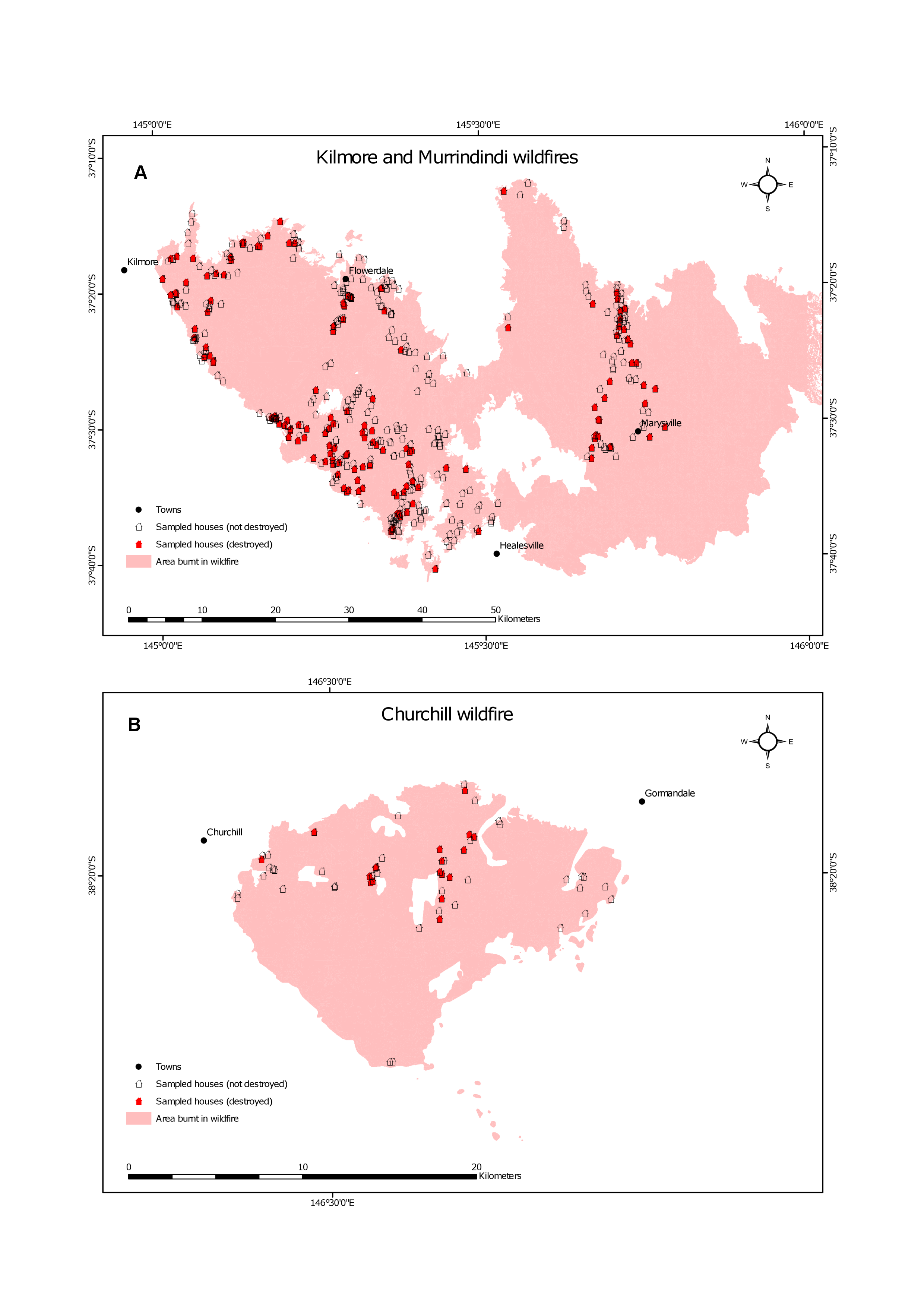

Supplement: Figure S1 — Houses sampled in (A) the Kilmore East Murrindindi wildfires and (B) the Churchill wildfire. Sampled houses that were intact (clear houses) and destroyed (solid red houses) after the wildfires are illustrated. (TIF) [file pone.0029212.s001.tif]
