# Supplementary material for: Land Management Practices Associated with House Loss in Wildfires
Source: PLoS One. 2012 Jan 18;7(1):e29212. doi: 10.1371/journal.pone.0029212 (PMC3260958; doi:10.1371/journal.pone.0029212)
Supplement: Table S1 — Potential explanatory variables recorded for each sampled house. (DOC) [file pone.0029212.s002.doc]

| **Variable** | **Definition** |
| --- | --- |
| Slope | Calculated in degrees using ArcMap and a DEM with a 10m×10m resolution. We calculated mean slope across a 100mx100m window centred on each house. |
| Aspect | Calculated as degrees from north in ArcMap from a DEM with a 10mx10m resolution. We calculated mean degrees from north across a 100mx100m window centred on each house and reclassified into a factor with four levels (N >315°-45°, E >45°-135°, S >135°-225°, W >225°-315°). |
| Topographic position | Calculated at each house as one of seven levels (1=ridge to 7=valley bottom) using a modified algorithm of Summerell et al. [38] on the 10m×10m DEM re-sampled to 100mx100m because of computational difficulties. |
| Buildings | The number of buildings (excluding circular water tanks) visible on the imagery intersecting a circle with a radius of 40m from the centroid of each house. |
| % cover of trees and shrubs | Visual estimate of % woody vegetation within a circle with a radius of 40m from the centroid of each house using the pre-fire imagery. This estimate was verified against digitised data (see text for explanation). |
| Vegetation type (planted and remnant) | A visual assessment of whether woody vegetation within a circle with a radius of 40m from the centroid of each house was predominantly planted or remnant using the pre-fire imagery. The features of trees and shrubs that were indicative of their origin were: crown texture, size, shape and arrangement relative to trees in nearby remnant vegetation. |
| Distance to nearest tree or shrub | Distance from each house to nearest tree or shrub (m) visible on the pre-fire imagery from the nearest edge of each house in any direction measured manually in a Geographical Information System (GIS) using the pre-fire imagery. |
| Distance upwind to nearest of trees or shrubs | Distance from each house to nearest group of ≥2 trees or shrubs (or one tree if its canopy was ≥10m wide) from the edge of the house in the upwind direction measured manually in a GIS using the pre-fire imagery. |
| Distance upwind to nearest block of trees | Distance from each house to nearest block of trees ≥50m wide at the narrowest point from the edge of the house in the upwind direction measured manually in a GIS using the pre-fire imagery. |
| Distance upwind to mapped cleared land | Distance from each house to nearest area without woody vegetation (as mapped in the NV2005_EXTENT GIS raster provided by DSE) in the upwind direction. |
| Adjacent forest type upwind | The nearest mapped floristic forest type (12 levels) (FTYPE in the VEG_91 GIS shape file provided by DSE) to each house in the upwind direction. |
| Adjacent forest structure upwind | The nearest mapped forest type structure (open forest, woodland, non-forest) (STRUCTURE in the VEG_91 GIS shape file provided by DSE) to each house in the upwind direction. |
| % cleared upwind | % mapped woody vegetation calculated along a transect in the upwind direction from each house to the 2009 wildfire boundary using the NV2005_EXTENT GIS raster provided by DSE. |
| Amount of land not burnt for ≤5 years upwind | Amount (m) of land from each house that was not burnt for ≤5 years prior to 2009 (as mapped in the PROD_FIRE_LASTBURNT100 layer provided by DSE) measured in the upwind direction. |
| % burnt ≤5 years ago upwind | % of landscape burnt ≤5 years ago calculated along a transect from each house to the 2009 wildfire boundary in the upwind direction using the PROD_FIRE_LASTBURNT100 GIS shape file provided by DSE. |
| Amount of land not burnt for >5-10 years upwind | Amount (m) of land from each house that was not burnt for >5-≤10 years prior to 2009 (as mapped in the PROD_FIRE_LASTBURNT100 shape file provided by DSE) measured in the upwind direction. |
| Upwind % burnt 5-10 years ago | % of landscape burnt >5-≤10 years ago calculated along a transect from each house to the 2009 wildfire boundary in the upwind direction using the PROD_FIRE_LASTBURNT100 GIS shape file provided by DSE. |
| Upwind amount unlogged | Amount (m) of land from each house that is unlogged in the previous 30 years (as mapped in the LASTLOGGED GIS shape file provided by DSE) in the upwind direction. |
| Upwind % logged | % of landscape logged in the 30 years prior to 2009 calculated along a transect from each house to the 2009 wildfire boundary in the upwind direction using the using the LASTLOGGED GIS shape file provided by DSE. |
| Upwind amount of private land | Amount (m) of land from each house that is not a public land tenure (as mapped in the PLM100 GIS shape file provided by DSE) in the upwind direction. |
| Upwind amount of land not National Park | Amount (m) of land from each house that is not National Park (as mapped in the PLM100 GIS shape file provided by DSE) in the upwind direction. |
| Upwind amount of land not State Forest | Amount (m) of land from each house that is not State Forest (as mapped in the PLM100 GIS shape file provided by DSE) in the upwind direction. |
| Upwind distance to wildfire boundary | Distance (m) from each house to the 2009 wildfire boundary (as mapped in the FIRE_SEV09 GIS shape file provided by DSE) in the upwind direction. |
